# Agency, access, and *Anopheles*: neighborhood health perceptions and the implications for community health interventions in Accra, Ghana

**DOI:** 10.3402/gha.v8.26492

**Published:** 2015-05-20

**Authors:** Marta M. Jankowska, Justin Stoler, Caetlin Ofiesh, David Rain, John R. Weeks

**Affiliations:** 1 Department of Family Medicine and Public Health, University of California San Diego, San Diego, CA, USA; 2 Department of Geography and Regional Studies, University of Miami, Coral Gables, FL, USA; 3 Department of Public Health Sciences, Miller School of Medicine, University of Miami, Miami, FL, USA; 4 Department of Geography, The George Washington University, Washington, DC, USA; 5 Department of Geography, San Diego State University, San Diego, CA, USA

**Keywords:** Africa, Ghana, neighborhood effects, health perceptions, malaria, community intervention, health-care access

## Abstract

**Background:**

Social and environmental factors are increasingly recognized for their ability to influence health outcomes at both individual and neighborhood scales in the developing urban world. Yet issues of spatial heterogeneity in these complex environments may obscure unique elements of neighborhood life that may be protective or harmful to human health. Resident perceptions of neighborhood effects on health may help to fill gaps in our interpretation of household survey results and better inform how to plan and execute neighborhood-level health interventions.

**Objective:**

We evaluate differences in housing and socioeconomic indicators and health, environment, and neighborhood perceptions derived from the analysis of a household survey and a series of focus groups in Accra, Ghana. We then explore how neighborhood perceptions can inform survey results and ultimately neighborhood-level health interventions.

**Design:**

Eleven focus groups were conducted across a socioeconomically stratified sample of neighborhoods in Accra, Ghana. General inductive themes from the focus groups were analyzed in tandem with data collected in a 2009 household survey of 2,814 women. In-depth vignettes expand upon the three most salient emergent themes.

**Results:**

Household and socioeconomic characteristics derived from the focus groups corroborated findings from the survey data. Focus group and survey results diverged for three complex health issues: malaria, health-care access, and sense of personal agency in promoting good health.

**Conclusion:**

Three vignettes reflecting community views about malaria, health-care access, and sense of personal agency in promoting good health highlight the challenges facing community health interventions in Accra and exemplify how qualitatively derived neighborhood-level health effects can help shape health interventions.

The past two decades have witnessed a surge of literature on the relationship between neighborhoods and health. The methodologies for understanding how context and place influence health are increasingly being applied in developing urban contexts (1–3). Such research has generated insights for neighborhood-level interventions and initiatives concerning a wide range of health issues in sub-Saharan Africa, such as intimate partner violence (4), maternal health (5), and tuberculosis (6). However, the use of traditional survey tools and modeling techniques for measuring neighborhood contextual effects on health may result in confusing or counterintuitive results due in part to the complexities of developing urban systems. One source of insight into neighborhood health effects is a community's own health perceptions, particularly when coupled and compared with individual and household survey results. Health perceptions may be especially helpful when expected relationships between place and health are not observed and may provide alternative theories for contextual influences on health. Furthermore, local health perceptions may include key aspects for planning health interventions at the neighborhood level, which may not be apparent in survey results or quantitative models.

Traditionally, the urban transition has been associated with better health, increased wealth, and improved access to amenities such as water and electricity. Yet public infrastructure and government programs are not keeping pace with population growth in a rapidly urbanizing developing world, resulting in variable landscapes characterized by inequalities in housing, socioeconomic status, environmental conditions, and health (7, 8). Ghana is part of this urbanizing trend and is currently a majority urban country. The capital city, Accra, is located on West Africa's southern coast along the Gulf of Guinea. The Accra Metropolitan Area population is estimated at 1.8 million from the 2010 census; however, the city is part of a rapidly expanding dense urban region known as the Greater Accra Metropolitan Area, which has a population of 4.0 million (9). In Accra, both the lack of enforced zoning regulations and the shortage of affordable housing contribute to a ‘spatially messy’ landscape (10). In many high-income (HI) neighborhoods, it is common to see small shacks, kiosks, and other informal structures filling in gaps between formal developments. In low-income (LI) areas, some residents achieve higher incomes as chieftains, landlords, or agents – especially those who erect structures in informal settlements – and local power dynamics keep these entrepreneurs in communities that they could otherwise afford to leave. In both of these examples, residential in-fill causes significant heterogeneity of socioeconomic status and, by proxy, variation in health outcomes typical of the developing world (11).

Individual-level predictors for health can become complicated by the overwhelming environmental burden of living conditions such as lack of sanitation and poor air quality (12–16). Neighborhood characteristics, such as access to nutrition or medical care, can also be protective of health, as demonstrated by the suspected role of sachet drinking water in reducing childhood diarrhea in Accra's slums (17). Problematically, many statistical modeling techniques utilized in studies of neighborhood effects control for heterogeneity (variance), effectively discounting the complex relationships between environment and health that may be of particular interest (18). These issues have surfaced in several ecological studies related to socioeconomic status, health behaviors, health outcomes, and environmental hazards in Accra, resulting in conflicting, counterintuitive, or spatially inconsistent findings (1, 17, 19–26).

In order to untangle and better understand neighborhood effects, it is becoming increasingly necessary to consider unique conceptualizations of how place might impact health. Studies on perceptions and beliefs about disease and health behaviors are one possible method of teasing out important interactions between place and health by assessing how individuals view their neighborhood as interacting (or not) with their health. For example, Eyles et al. (27) interviewed participants in Hamilton, Ontario, regarding environmental risk perceptions and found that scale was not simply a nested hierarchy of types of hazards (contexts), but that each scale represented a different level at which individuals experienced links between their environment and health, with different actions taken at different scales. This finding is an important reminder that while environmental risks may operate in a hierarchical fashion, how individuals respond to those risks may not be hierarchical.

Personal beliefs about disease are important driving factors of mortality and morbidity reduction in the developing world (28). Furthermore, how people share and apply that knowledge within their social and neighborhood networks can shape health knowledge and disease outcomes (29). Incorporating individual and community perceptions of health into neighborhood effects studies can be an important input for statistical models, as perceptions can be more influential than factual information in driving health-related behaviors (30, 31). Research concerning perceptions about place and health in developing urban environments is scarce, particularly studies that compare and contrast perceptions from different contextual backgrounds within a city. Unobservable context – such as communal lack of knowledge about a health risk, or cultural factors that inadvertently circumscribe healthy behaviors – is often difficult to capture in health surveys that primarily inventory household characteristics and symptoms and that categorize health perceptions on Likert-type scales. Methods utilized in community-based participatory research that are aimed at rooting out perceptions, for example focus groups, can assist in shaping survey tools, make sense of model results, help increase understanding of how residents view their neighborhood and environment as a potential influence on their health, and lead to better quantitative measures of neighborhood context (32, 33). Better measurement, in turn, may lead to a better understanding of how to plan and execute neighborhood-level interventions.

In this study, focus group sessions assist in teasing out community perceptions of neighborhood-level health effects. The paper emphasizes the importance of focus group viewpoints from neighborhood residents as key catalysts for understanding conflicting or difficult-to-interpret survey results, and vignettes are used to explore how these viewpoints may be important elements for planning neighborhood-level health interventions in Accra. We compare a descriptive analysis of survey data gathered from 2,814 women in the 2008–2009 Women's Health Study of Accra, Wave II (WHSA-II, a broad inventory of urban women's health status), with health themes derived from 11 focus groups conducted across a socioeconomically stratified sample of comparable neighborhoods in 2010. Focus group questions center on topics related to socioeconomic indicators, housing, neighborhood health, access to care, and the extent to which residents perceived a sense of community in their neighborhood. Three vignettes synthesize survey and focus group results by reflecting on community views about malaria, health-care access, and sense of personal agency in health outcomes; these discussions illustrate how health perceptions can supplement and inform analysis of the household survey data by illuminating how individuals view their interaction with the local environment. The vignettes also provide examples of how resident perceptions vary from neighborhood to neighborhood, with implications for health interventions at the neighborhood level. The results have helped to inform our interpretation of neighborhood influence on health in Accra, and they yield implications for both environmental health research and health interventions.

## Methods

**Fig. 1:**
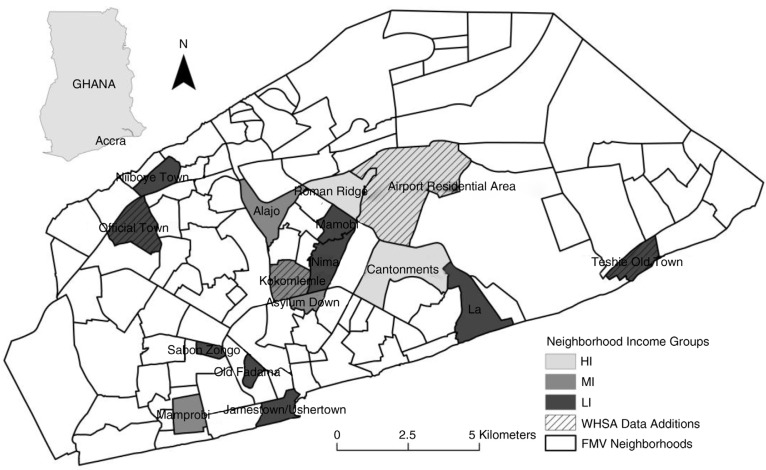
Field modified vernacular neighborhoods for the Accra Metropolitan Area displaying focus group neighborhood locations and supplemental status-equivalent neighborhoods from WHSA-II shaded by income level.

The focus group sample was selected from a set of neighborhood boundaries developed in previous research known as field modified vernacular (FMV) neighborhoods (Fig. 1). These boundaries were created through an integrative fieldwork process that attempted to encapsulate local resident perceptions, man-made and natural barriers, and socioeconomic milieu into a set of 108 neighborhood boundaries (34).

The initial wave of the WHSA was conducted in 2003 as a collaboration between the Institute of Statistical, Social and Economic Research (ISSER), University of Ghana, and Harvard School of Public Health and consisted of detailed health and household interviews of a representative sample of almost 3,200 women aged 18 and over (35). The second wave (WHSA-II) was a follow-up study of 2,814 women conducted in 2008–2009. WHSA-II consisted of 25 sections of questions covering topics such as the women's general characteristics, general health, self-care, pain and discomfort, community role, household characteristics, reproductive health, participation in national health insurance, and basic anthropometric indicators. Survey instruments and results are available from ISSER (36) and Darko et al. (37).

After implementing the WHSA-II, the research team conducted 11 focus groups in a socioeconomically diverse selection of FMV neighborhoods (Fig. 1, shaded by income level), with the goals of learning residents’ perceptions of their health and neighborhood surroundings and discerning similarities and differences in perspectives between neighborhoods. LI neighborhoods were oversampled to reflect the distribution of women in the WHSA-II. Of the eleven neighborhoods, seven had already been sampled during the WHSA-II survey, while four were deliberately chosen because they were not part of that survey, thus allowing us to compare perspectives from new areas. For each of the four neighborhoods that were not surveyed during WHSA-II, a neighborhood of similar socioeconomic ranking was selected for comparison based on expert knowledge of the city. These pairings are highlighted in Fig. 1: Teshie Old Town (for Old Fadama), Kokomlemle (for Asylum Down), Airport Residential (for Roman Ridge), and Official Town (for Nii Boye Town).

Once the target neighborhoods were identified, focus groups were arranged and recruited by two research assistants from the University of Ghana (both Accra residents), who contacted the leadership of each neighborhood – the Assemblyperson and/or the local chief – and worked with community groups to organize participants. All recruitment was conducted verbally and based on convenience and snowball sampling. Not all individuals invited were able to attend due to availability or willingness to participate. Refusal rates were not recorded as individuals attended at their convenience, but were generally low. Focus groups were conducted at diverse venues, including a church, chop bar, school, and private residence.

The focus group discussion research team consisted of four researchers (authors MJ, JS, CO, and DR) and the two research assistants from the University of Ghana. At least three of the four researchers were present for all focus groups. Author DR served as the project lead in the field and served as primary facilitator, leading semistructured focus group discussions with a standardized discussion script. Authors MM, JS, and CO took notes, asked follow-up questions, and reached out to quieter focus group members to solicit opinions and participation in discussion. Each focus group lasted between 1 and 2.5 hours, inclusive of question and answer time at the conclusion of each session. The majority of the discussions were conducted in English, with the research assistants providing translation as needed. The focus group in Old Fadama was the sole exception; it was conducted entirely in Hausa and translated by the research assistants to the research team.

The number of participants ranged from 8 to 17 (dependent on the recruiting success of local community groups) and varied by gender (Table 1). Both men and women were included in focus groups to gather diverse neighborhood perceptions and supplement the WHSA-II female-only perspective. Some groups (Jamestown/Ushertown, Alajo, Cantonments, and Roman Ridge) were predominantly composed of one gender or another (Table 1); however, in each case the research team made efforts to ensure that the minority gender had ample opportunities to be heard. Age and socioeconomic status of participants was not collected in order to respect their privacy, but the focus groups included a mix of young and older adults. The research team's perceptions of focus group dynamics and interactions were very positive. Although there were numerous outspoken individuals, no focus group was dominated by any one individual, and throughout the sessions the team made efforts to ensure that everyone had a chance to speak.

**Table 1:** Focus group neighborhood names, general economic levels, and compositions

Neighborhood	Economic level	Number of participants	Gender balance (men/women)	Surveyed in WHSA-II
Nii Boye Town	Low	8	6/2	No
La	Low	12	8/4	Yes
Nima/Mamobi	Low	13	7/6	Yes
Jamestown/Ushertown	Low	11	10/1	Yes
Sabon Zongo	Low	11	8/3	Yes
Old Fadama	Low	12	9/3	No
Asylum Down	Medium	8	4/4	No
Alajo	Medium	11	1/10	Yes
Mamprobi	Medium	14	3/11	Yes
Roman Ridge	High	17	16/1	No
Cantonments	High	10	9/1	Yes

The focus group meetings covered a number of topics pertaining to the neighborhood's environmental and social milieu, as well as resident perceptions of environment and health. Topics were intended to correspond to areas of study in the WHSA survey. Subjects of discussion included the following: housing and tenure, rent and landlords, types of jobs available, movement of individuals in and out of the neighborhood throughout the day, drinking water and household water access, quality and rationing of water, access to toilets and sanitation, solid and liquid waste disposal, sanitation-related health threats, access to health clinics and pharmacies, perceived levels of health in the neighborhood, perceived neighborhood health compared with other neighborhoods, environmental factors influencing health, top neighborhood health threats, vulnerable populations, local political empowerment, neighborhood sense of community, and presence of neighborhood organizations. (The complete focus group script is available from the authors upon request.)

The focus group discussions were recorded and transcribed, and later amalgamated using the written notes taken by the researchers during the sessions. The structured focus group script allowed for easy categorization of topical areas of discussion. The content for each topic was summarized using a general inductive approach (38), focusing on key emergent themes for each topic. Themes were then compared across income categories of the focus groups. Focus group results for each neighborhood were compared with similar indicators from the second wave of the WHSA-II.

We summarized WHSA-II responses from 886 women (564, 297, and 25 from LI, middle-income [MI], and HI neighborhoods, respectively) across the 11 neighborhoods and compared them with themes derived from the focus groups. It should be noted that participants from HI neighborhoods were not necessarily HI earners *per se*, but were sometimes house help or workers employed by high-earners who also lived in the HI neighborhood and enjoyed community amenities. We tested for statistically significant (*α=*0.05) pairwise differences between the three income groups (LI, MI, HI) using the Dunnett T3 test of means to account for unequal variances among the groups; all quantitative analysis was performed using IBM SPSS Statistics version 21.

After comparing quantitative and qualitative findings across income groups, we expand upon the most substantive findings in a series of discussion-based vignettes. The vignettes offer further synthesis of results and elaborate upon the implications for community-level health interventions.

## Results

### Housing and socioeconomic indicators

Table 2 highlights some of the key descriptive characteristics from the WHSA-II for women in LI, MI, and HI neighborhoods, which correspond to the same three categories of neighborhoods in Table 1 in which our focus groups were conducted (detailed summary statistics for the full WHSA-II sample are available in the Technical Publication report) (36). In previous studies of Accra households, households in well-established LI neighborhoods tended to comprise a larger percentage of compound housing with longer-term occupants and inferior water and sanitation access (19, 39, 40). These patterns are generally evident in the WHSA-II data, where we observed expected statistically significant differences between households grouped by LI, MI, and HI neighborhoods for the respective percentages of compound housing (85, 61, 24%), self-contained housing (14, 39, 72%), piped water access inside the home (38, 51, 64%), toilet in home access (15, 43, 80%), and household sewer connection (6, 17, 48%). In addition, households in LI neighborhoods were statistically significantly different from those living in MI and HI neighborhoods for years of tenure in the home (26 vs. 18 and 13 years, respectively), piped water access in the home (38 vs. 51 and 64%), primary use of public toilets (54 vs. 19 and 16%), and access to a trash collection service (28 vs. 39 and 60%).

**Table 2:** Descriptive household and individual characteristics from WHSA-II summarized by neighborhood income level

Characteristic	Low income	Middle income	High income
*Household level*			
Housing type (%)			
Self-contained/separate	14.4^a^^,^^b^	39.1^b^	72.0
Compound	84.9^a^^,^^b^	60.6^b^	24.0
Housing tenure (%)			
Own	25.8^a^^,^^b^	37.6^b^	8.0
Rent	38.8	36.9	24.0
Rent-free	35.2^a^^,^^b^	25.1^b^	68.0
Years in house	26.2^a^^,^^b^	17.9	12.8
Primary water source (%)			
Pipe in home	37.8^a^^,^^b^	50.7	64.0
Pipe outside home	46.3^b^	39.2^b^	16.0
Sachet water	8.3	8.1	20.0
Primary toilet access (%)			
Toilet in home	14.7^a^^,^^b^	43.4^b^	80.0
Public toilet	54.2^a^^,^^b^	19.3	16.0
Trash collection service (%)	27.7^a^^,^^b^	38.7	60.0
Sewer connection in home (%)	6.4^a^^,^^b^	17.2^b^	48.0
*Individual level*			
Employment (%)			
Informal	52.0	47.5	52.0
Professional	9.0^a^^,^^b^	14.8	36.0
Primary health-care source (%)			
Clinic	61.8	60.5	63.6
Hospital outpatient	15.9	22.4	13.6
Pharmacist	12.8^a^	6.5	13.6
Top neighborhood health concern (%)			
Malaria	15.1	15.8	8.0
Environment	4.1^b^	5.4^b^	0.0
Other communicable disease	4.3	2.0	8.0
Non-communicable disease	3.9	3.0	4.0
Perceived source of malaria (%)			
Mosquitoes	62.8^a^^,^^b^	79.1	88.0
Environment	12.8	8.8	4.0
Unsure	24.1^a^^,^^b^	11.8	8.0
Voted in 2004 (%)	92.9	90.6	96.0
Registered to vote in 2008 (%)	90.4^a^^,^^b^	97.0^b^	100.0

a *p<*0.05 for pairwise comparison with middle income group.

b *p<*0.05 for pairwise comparison with high income group.

Pairwise comparisons utilize Dunnett T3 test of means to account for unequal variances.

We observed unexpected statistically significant differences in home ownership (26, 38, 8%) and rent-free living (35, 25, 68%), which reflects a mix of lower-income individuals living in HI neighborhoods. These particular neighborhoods exhibited no significant differences by income level for percentage of renters or for reliance on sachet drinking water. We observed fewer differences between income groups at the individual level. The percentage of women professionally employed was only statistically significantly lower for women of LI neighborhoods compared with MI and HI areas (9 vs. 15 and 36%, respectively), and there were no significant differences across income groups for informal employment.

Table 3 summarizes key focus group responses by neighborhood. Themes that emerged from the housing and socioeconomics discussions generally supported results from the WHSA-II. We observed similar trends across neighborhood income groups in housing type, rent amounts, and housing tenure; neighborhood employment and commuting patterns; and access to infrastructure and city services such as water, sewers, and solid waste disposal.

**Table 3:** Summary of focus group themes by neighborhood socioeconomic status

Focus group theme	Low income	Middle income	High income
Dominant housing type	Compound living; mix of renting/owning single family units; shack ownership	Compound living; renting of single family units	Single family units, often government-owned
Average monthly rent and required deposit	6–8 GHC for shared room; 15–40 GHC for single room with 2–3 years’ rent paid in advance	15–45 GHC for single room, with 2–3 years’ rent paid in advance	1,000–1,500 USD for flat or single family home; 1–3 years paid in advance
Use of rental agents to find housing	Usually informal agents used; occasionally no agents involved	Usually informal agents used; occasionally formal agents hired	Agents rarely used
Typical employment patterns	Majority informal trades; some formal employment	Majority informal trades; formal employment increasingly common	Formal employment, particularly government or military sectors
Daily commuting patterns for employment	Most working residents leave neighborhood for employment; varying flows of workers coming into the neighborhood for work	Most working residents leave neighborhood for employment; varying flows of workers coming into the neighborhood for work	Most workers leave to work in other parts of the city, hired help comes in during the day
Household water source	Mix of public and private taps; water is occasionally sold to others	Mostly private taps; water is often sold to others	Private taps
Degree of neighborhood water rationing	Varying degrees of rationing	No rationing	No rationing
Drinking water source	Primarily sachet water, some tap water	Primarily sachet water, some tap water	Sachet water and tap water equally used
Primary toilet source	Public KVIP	Mix of public KVIP and private toilets	Private toilets
Solid waste disposal options	Some collection services and use of public dumps; dumping in gutters common	Some collection services; dumping in gutters common	Weekly collection services
Primary health-care source	Public clinics, pharmacies, drug vendors	Public clinics, pharmacies, occasionally private clinics	Mix of public and private clinics; sometimes pharmacies
Primary health concerns	Malaria; diarrheal diseases	Malaria; diarrheal diseases	Malaria; stress
Perceived health of neighborhood residents compared to other similar areas	Varying perceptions (less healthy, as healthy as, and healthier than similar neighborhoods)	As healthy as similar neighborhoods	As healthy or healthier than similar neighborhoods
Perceived impact of the environment on health	Mixed perceptions of personal hygiene versus environmental influence on health	Personal hygiene perceived as more important than environmental influences, but role of environment acknowledged	Personal hygiene perceived as more important than environmental influences
Perceived political voice	Mixed degrees of satisfaction with local representation; varying numbers of local organizations and NGOs	General dissatisfaction with representation; few or no local political organizations	Mixed degrees of satisfaction with local representation; few or no organizations
General sense of community	Generally strong, except where there are significant numbers of new migrants	Moderate; friendliness among neighbors, but little sense of larger community within neighborhood	None, not even between neighbors

KVIP: Kumasi ventilated improved pit.

### Health, environment, and neighborhood perceptions

There was less agreement observed between self-reported WHSA-II and focus group results on issues of personal health maintenance, access to health care, role of the environment, and perceptions of neighborhood sense of community and social or political engagement. When asked about their most pressing health concern for their neighborhood, women in the WHSA were most concerned about mosquitos and malaria, but there were no differences between neighborhood categories. Women in LI and MI neighborhoods were significantly more likely to cite ‘the environment’ as their top neighborhood health concern (4 and 5%, respectively) than women in HI areas (0%), but there was again no evidence of differences for other communicable diseases or non-communicable diseases between neighborhood categories.

Across all focus groups, regardless of any other factors, participants overwhelmingly perceived malaria as the number one health issue in their neighborhoods. This finding is in agreement with the WHSA-II, but the importance of this issue to residents is underrepresented in the WHSA-II; less than 20% of any income group in the WHSA-II indicated malaria as a primary concern, in stark contrast with the near-unanimous voicing of concern over malaria in the focus groups. In the WHSA-II, women were significantly less likely to attribute malaria to mosquitoes in LI neighborhoods (63%) compared with MI and HI areas (79, 88%), or to simply not know the source of malaria (24 vs. 12 and 8%). Environment was listed as a source of malaria by some women, although in the focus groups it became clear that there is a significant lack of education and understanding about where malarial mosquitos originate.

There were no significant differences across income groups in the proportion of women seeking primary health care at clinics and hospital outpatient facilities, although women from LI neighborhoods were significantly more likely to rely on primary care from pharmacists (13%) than women in MI neighborhoods (7%), but not from HI areas (14%). In the focus groups we learned that access and choice of care is more complicated than the household survey would suggest. Whereas the WHSA-II showed similar levels of clinic usage, the focus groups revealed a disaggregation of clinic usage into public and private clinic patronage. Additionally, focus groups results indicated a strong reliance of LI and MI neighborhood residents on pharmacies and informal drug sellers as a first line of defense in illness, particularly malaria.

Finally, an important theme that emerged from the focus groups that was not apparent from the WHSA-II survey results was the relative perceived merit of individual agency and the neighborhood environment in preserving good health. Participants from LI areas voiced strong associations between the environment and their personal health. Although focus group participants in LI areas tended to have a mix of perceptions about the relative importance of the environment and personal hygiene, this mix increasingly tilted toward an emphasis on personal hygiene when focus groups were conducted in MI and HI areas.

## Discussion

Whereas focus groups did allow for deeper probing into residents’ experiences with city services and issues such as renting from agents or multi-year prepayment of rent, the themes that emerged from the housing and socioeconomics discussions generally supported results from the WHSA-II. This consistency lends validity to the survey results and supports the use of the survey as a representative tool for key housing and socioeconomic indicators in the city. However, we witnessed less agreement with WHSA-II survey results on complex health issues and observed a more nuanced range of opinions and attitudes. The following three vignettes exemplify how the links between certain types of health perceptions and health behaviors can be difficult to isolate in household surveys but may have important implications for neighborhood-level health interventions.

### Vignette 1 – Malaria: misplaced perceptions of a real threat

The significance of malaria as a primary health threat cuts across communities of all socioeconomic levels. Whereas the perception of solid waste disposal as a driver of public health varied between higher- and lower-income neighborhoods, the linkage of malaria to sanitation inadequacy was a persistent theme. Residents of all communities associated excess garbage and clogged drains with the propagation of mosquitoes and, therefore, heightened risk of malaria, yet expressed less concern over diseases such as typhoid fever that are much more likely to be sanitation-related. This misconception was not reflected in the survey but could be addressed with a more specific question such as, where do the mosquitoes that cause malaria come from? This linkage stands out as the most prevalent health misperception we encountered, particularly in light of malaria's heavy morbidity burden in Ghana.

Several species of mosquitoes with public health implications have been documented in Accra, with *Culex* species being dominant (41, 42). *Culex* mosquitoes are well-adapted to breed in highly polluted aquatic environments and serve as a vector for several diseases including West Nile virus, filariasis, and Japanese encephalitis. The primary vector for malaria in Ghana, *Anopheles gambiae*, typically breeds in clean, still, or slow-moving water near vegetation, such as is found in irrigation canals, though it utilizes a range of habitats (43). Despite growing evidence that *A. gambiae* is adapting to polluted water in sub-Saharan Africa (42, 44–46), the piles of solid waste that accumulate due to lack of sanitation services are unlikely to provide significant *Anopheles* breeding habitat, though pooling water in clogged drains may warrant further examination. By contrast, urban agriculture has been shown to support *Anopheles* breeding habitat and increase malaria risk for nearby residents in Accra (47), even among populations with higher socioeconomic status (24). No focus group participants in any neighborhood vocalized a perceived health risk from proximity to agriculture, even with prompting.

The difficulty in properly diagnosing malaria without a laboratory blood test is well known, and most cases in sub-Saharan Africa remain self-diagnosed and treated at home (48). The primary symptoms of malaria overlap with many infectious diseases, making differential diagnosis difficult (49, 50). Because the presence of fever is often presumptively treated as malaria, even when malaria may not be circulating (51), the true burden of diseases such as typhoid, influenza, and dengue fever are likely understated in Accra (52). Focus group participants frequently cited typhoid as an important health risk linked to water and sanitation inadequacies in their communities, and there has been more media attention on water-borne illness and hand washing in light of a local cholera outbreak in Accra in January 2009 (a subsequent outbreak occurred in early 2011). Participants expressed little concern over other febrile diseases, and awareness of viral hemorrhagic fevers such as yellow fever and dengue fever was virtually absent despite the longstanding presence of vector *Aedes aegypti* in Accra (53, 54). Community perception of a health risk through conflation of mosquito and sanitation issues, although erroneous with respect to malaria, is likely a proxy for risk of other febrile conditions that plague some neighborhoods yet go relatively undetected by health-care facilities that are focused on malaria. When planning a malaria intervention, should it matter if community perceptions of health risks are accurate or not?

Previous studies have demonstrated that knowledge of malaria transmission and vectors is associated with better adherence to vector control interventions (55, 56). Yet the common knowledge held by individuals may influence educational processes, where the generation of new knowledge often results from a relationship between common and scientific knowledge (57). What if a community correctly perceived a high malaria burden, but was wrong about the source? A traditional bed-net intervention might reduce true malaria cases, but would be unlikely to reduce the misdiagnosed malaria cases that were attributable to other water- or vector-borne diseases. If other febrile illnesses persisted, sustained bed-net usage might erode as households lost confidence in the value of the intervention. Conversely, what if a community overestimated its malaria burden, but correctly perceived inadequate sanitation as an important health threat? Although improvements to water and sanitation infrastructure might not directly affect the true local malaria burden, such cleanup efforts would likely reduce malaria overdiagnosis by reducing morbidity from other underlying causes. However, any subsequent malaria cases in the community that were confirmed by blood test at a clinic might again, depending on prevalence, undermine the perceived efficacy of the intervention. The takeaway message is that a perceived neighborhood effect on health, such as degree of sanitation adequacy in a community, may or may not be linked to the community's primary health concerns or individual-level risk factors, yet could be crucial for the proper design of health interventions.

### Vignette 2—Access to health care and decision-making: the clinic conundrum

Accessibility to health care is commonly reasoned to exert considerable pressure on variability in health outcomes between neighborhood populations. Health-care access is often understood as either neighborhood density or individual distance metrics to formal institutions, which can be located and counted. In Accra, access to formal primary and secondary health care, computed as neighborhood density of health-care clinics, was not found to be related to neighborhood trends of under-five child mortality (23). In unpublished multilevel modeling results, this measure of access was also non-significant in predicting women's self-reported general health. In order to better understand these counterintuitive results, groups were asked ‘If people need health care, where do they go, and why do they choose to go there?’

Respondents for all groups were able to quickly and accurately identify numerous public and private health clinics located within or close to their neighborhoods, as well as the major hospitals located throughout the city. Spatial access to health care was not seen to be an issue for any of the groups, because health clinics were easily and affordably accessed by taxi or *tro-tro* (public minibus transport). Participants demonstrated more concern over temporal and monetary access. Public clinics were generally seen as a waste of time, but a necessity when money was tight. Six groups discussed the wait times for public clinics, which they all cited as being a day-long wait to be seen. The extensive wait times were perceived to be a difficult barrier if an individual had a job or if the health concern was pressing. Although private clinics were unanimously seen as better health treatment options with much faster service, many groups agreed that cost was often a prohibitive factor in seeking private clinic treatment. One MI group noted that, if a problem was serious enough and time was of the essence, even very poor people would find the money to be seen at a private clinic.

In contrast with the WHSA-II data indicating that approximately 60% of women go to local clinics for primary care, clinics (both private and public) were resoundingly *not* the first step in health-care-seeking behavior in all focus groups. The importance of pharmacies, traveling drug sellers, and natural healers in markets as a first line of treatment was emphasized in all focus groups except those in Roman Ridge and Cantonments, both HI neighborhoods. Self-medication without doctor or pharmacist consultation was mentioned by five focus groups, indicating strong personal agency in treating a variety of illnesses. Self-medication or medication through a pharmacist was also seen as a common way of treating malaria. These focus group findings reflect previous research concerning pervasive self-medication in Accra (58). Numerous groups in both LI and MI neighborhoods mentioned that a primary reason for self-medication of malaria is that clinics and pharmacies often tell individuals to try antimalarials for symptoms of fever or vomiting and to only return if the pills do not work.

The emphasis on pharmacies and self-medication, as well as ease of access to public and private health-care clinics, makes a strong case for the need for better measures and definitions of neighborhood health-care access. The focus groups emphasize that access may not be best defined by a distance or density measure, but rather by quality, cost, or wait time. Any measure of health-care access would also need to include pharmacies, because they are clearly thought to be a first line of defense against disease in almost all of the focus group discussions. The focus group results also have implications for intervention strategies that involve access to medications and related health aids. Although many pharmacies in Accra have trained staff with adequate medical supplies, many others are likely deficient in quality of medicines and knowledge (59). Considerable investment is placed in training, staffing, and supplying public and private health-care clinics throughout the city, whereas similar investments in pharmacies are lacking (60); all of this variation in resources may cause important neighborhood effects on health-seeking behaviors.

### Vignette 3 – Toilets and air pollution: personal agency vs. environmental context

Focus groups were asked about how their neighborhood environment affects overall health. Answers ranged from certainty that the environmental conditions of their neighborhood heavily impact their health, to beliefs that the environment has minimal influence on health and that personal hygiene practices protect against disease. Six focus groups reached a consensus that personal hygiene and individual agency are more important than the neighborhood environment in disease prevention, and some participants outright discounted the neighborhood environment having any effect on health. A more common approach was the acknowledgment that the environment could be harmful, but that personal choice, hygiene, and preparation could keep an individual healthy. One resident of a MI neighborhood noted, with respect to outbreaks, ‘You can tell who has prepared and who hasn't because [there are] those who don't get sick.’

In another MI neighborhood a participant suggested that, for example, having a Kumasi ventilated improved pit latrine in one's home rather than a toilet connected to the sewage system was a ‘personal choice’ rather than an environmental factor that might cause ill health. This is a poignant example of the tension between personal agency and the environmental conditions that individuals have little control over, particularly when compared to experiences in lower-income areas. In Jamestown/Ushertown, two bordering LI neighborhoods, residents overwhelmingly had no toilets in the home; space originally intended for toilets was converted into sleeping rooms as migration and natural growth led to severe overcrowding. Most residents used public toilets, which have been shown to be associated with higher rates of diarrhea, particularly for children (12). Numerous focus groups in the lower socioeconomic neighborhoods discussed the use of ‘flying toilets’ or ‘free range,’ in which residents defecate into a bag and toss the bag up onto a roof top or practice open defecation in open spaces and beaches. Participants’ awareness of these sanitation issues and the associated health threats to their communities are difficult to reconcile with the prevalent attitude that personal hygiene is enough to protect oneself from disease. This discrepancy may be explained by public health campaigns emphasizing individual-level actions such as hand washing (61) rather than neighborhood-level changes, but it may also be linked to cognitive factors. For example, recent findings show that women in many of Accra's slum communities rate their own health higher on average than do women in non-slum neighborhoods (21).

The Jamestown/Ushertown focus group was also the only group to report air quality as a possible source of ill health from the neighborhood environment, though this concern was secondary to other environmental concerns like sanitation and water. Regarding air pollution, one resident noted, ‘We are worried about it, but I don't think we care about it; we are used to it.’ Air pollution has been shown to be a considerable issue in Accra, especially in Jamestown/Ushertown (14, 15, 62). Respiratory issues comprise one of the largest health problems treated in clinics throughout Accra, and there is a significant spatial component associated with reporting and risks of respiratory disease (20, 63). However, there may be a mismatch between perceptions of agency expressed in the focus group and the clinical importance of individual versus neighborhood effects: a recent air pollution study in Accra demonstrated that community biomass use had a stronger association with household air quality than a household's own fuel choice (64).

If many individuals in Accra believe that personal agency and individual hygiene practices can protect against disease, the effects of a neighborhood-level health risk such as poor air quality may not fit into their perceptions of personal health. The non-prioritization of air pollution in the focus groups may indirectly speak to the need for interventions that address these types of neighborhood-level health risks. Additionally, there was clear variation among focus groups regarding the extent of residents’ belief in personal agency as a protective factor against environmental health risks. An intervention targeting environmental factors would likely fail in a community that held strong faith in personal behaviors, but might do better in a neighborhood that acknowledged the effects of environmental factors on health.

The focus group approach does come with attendant limitations such as potential moderator bias, the inhibition of responses from quieter attendees due to personality or community politics, and other response expectation biases. Due to the dynamic nature of focus groups, it is difficult to evenly cover each topic across groups. Some topics were covered more thoroughly than others depending on residents’ willingness to discuss certain issues, whereas in some focus groups (Old Fadama, Nii Boye Town, and La) certain topics were not discussed at all due to language barriers or time constraints. It can also be difficult to get a truly random demographic sample when using local community leaders to recruit focus group attendees, because both social networks and employment patterns can bias attendee invitation and availability.

The use of qualitative research to inform or help interpret qualitative findings is a longstanding and effective tool for health research. In the developing world, in an urban context where health outcomes are often the result of complex pathways, this type of approach can shed considerable light on counterintuitive or ambiguous survey and statistical findings; it can help us understand some of the neighborhood-level contextual influences on health. As health scientists apply neighborhood-level approaches for both research and intervention, focus groups and other qualitative methodologies may point to mediators and moderators of health that are not readily apparent in survey-based data, but are important to address when considering intervention strategies.

## Conclusions

We paired a subsample of household survey data with community-oriented focus groups in order to better interpret findings from each approach, while also noting the implications for public health interventions. Overall we observed a consistent picture of household and employment characteristics between the focus groups and WHSA-II. Agreement between methods is an encouraging finding that validates our previous survey work, lending support to the use of our socioeconomic and housing measures as applicable indicators in the urban Ghanaian context. We also observed some discrepancies in the interpretation of results from the survey and focus group for complex health issues, thereby highlighting the value of multiple methods in neighborhood health effects research. Our focus group discussions about health and the local environment allowed us to delve much deeper into these topics and uncover some promising avenues for better understanding complex health pathways, as well as engaging in neighborhood-level intervention strategies.

Malaria was the most common health concern, and findings from the focus groups highlight the potential for future studies to evaluate differences in malaria prevalence between neighborhoods with differential understandings of malaria ecology. New research has shown that the burden of other mosquito-borne diseases may be obscured by malaria misdiagnosis (65). New vector ecology studies that help us understand current mosquito breeding dynamics and adaptations may be crucial for improving diagnostics and surveillance of water- and vector-borne diseases.

In discussions about health care, the focus groups results indicated that, although individuals may have geographical access to health care, a better measurement of health-care access might include average wait time in a clinic, type and cost of the clinic, whether or not the clinic accepts insurance, and potential performance metrics for pharmacies in the area. Finally, in Vignette 3, we saw that individual- and neighborhood-level feelings about environmental effects on health will likely be a significant predictor of a successful neighborhood- (and individual-) level intervention. These types of community-level attitudes are likely reflected in other notions of cohesion, engagement, and trust in the political process, which are all connected to a community's belief in the prospect of change. Observed differences in perceived political voice and general sense of community from the focus groups portray a possible trend of increasing personal agency over health and a decreasing sense of community. The results offer insight into the relative levels of community capacity in neighborhoods of varying income levels, as well as the potential for success of public-private partnerships in the implementation of public health interventions, which may be more successful in lower-income communities.

Taken together, our analysis and vignettes suggest how (un)healthy behaviors may be shaped by perceptions and knowledge gaps, but perhaps more importantly how such perceptions can assist in our understanding and measurement of neighborhood effects on health and ultimately in designing health interventions for a developing urban center such as Accra.

## References

[1] Weeks JR, Getis A, Hill AG, Agyei-Mensah S, Rain D (2010). Neighborhoods and fertility in Accra, Ghana: an AMOEBA-based approach. Ann Assoc Am Geogr.

[2] Jankowska MM, Weeks J, Hill A, Stoler J (2013). Neighborhoods of health: comparing boundaries for measuring contextual effects on health in Accra, Ghana. Spatial inequalities: health, poverty and place in Accra, Ghana.

[3] Montgomery M, Hewett PC (2005). Urban poverty and health in developing countries: household and neighborhood effects. Demography.

[4] Uthman OA, Moradi T, Lawoko S (2009). The independent contribution of individual-, neighbourhood-, and country-level socioeconomic position on attitudes towards intimate partner violence against women in sub-Saharan Africa: a multilevel model of direct and moderating effects. Soc Sci Med.

[5] Stephenson R, Baschieri A, Clements S, Hennink M, Madise N (2006). Contextual influences on the use of health facilities for childbirth in Africa. Am J Public Health.

[6] Harling G, Ehrlich R, Myer L (2008). The social epidemiology of tuberculosis in South Africa: a multilevel analysis. Soc Sci Med.

[7] Montgomery MR (2009). Urban poverty and health in developing countries. Popul Ref Bureau.

[8] Rice J (2008). The urbanization of poverty and urban slum prevalence: the impact of the built environment on population-level patterns of social well-being in the less developed countries. Res Sociol Health Care.

[9] Ghana Statistical Service (2013). 2010 population and housing census. Accra, Ghana.

[10] Grant R (2009). Globalizing city: the urban and economic transformation of Accra, Ghana.

[11] Graham E, Boyle P, Curtis S, Moore E, Boyle P, Curtis E, Graham E, Moore E (2004). Health geographies in the developed world. The geography of health inequalities in the developed world: views from Britain and North America.

[12] Boadi KO, Kuitunen M (2005). Childhood diarrheal morbidity in the Accra metropolitan area, Ghana: socio-economic, environmental and behavioral risk determinants. J World Health Popul.

[13] De Snyder VNS, Friel S, Fotso JC, Khadr Z, Meresman S, Monge P (2011). Social conditions and urban health inequities: realities, challenges and opportunities to transform the urban landscape through research and action. J Urban Health.

[14] Dionisio KL, Rooney MS, Arku RE, Friedman AB, Hughes AF, Vallarino J (2010). Within-neighborhood patterns and sources of particle pollution: mobile monitoring and geographic information system analysis in four communities in Accra, Ghana. Environ Health Perspect.

[15] Dionisio KL, Arku RE, Hughes AF, Vallarino J, Carmichael H, Spengler JD (2010). Air pollution in Accra neighborhoods: spatial, socioeconomic, and temporal patterns. Environ Sci Technol.

[16] Vlahov D, Freudenberg N, Proietti F, Ompad D, Quinn A, Nandi V (2007). Urban as a determinant of health. J Urban Health.

[17] Stoler J, Fink G, Weeks JR, Otoo RA, Ampofo JA, Hill AG (2012). When urban taps run dry: sachet water consumption and health effects in low income neighborhoods of Accra, Ghana. Health Place.

[18] Merlo J, Ohlsson H, Lynch KF, Chaix B, Subramanian SV (2009). Individual and collective bodies: using measures of variance and association in contextual epidemiology. J Epidemiol Community Health.

[19] Arku G, Luginaah I, Mkandawire P, Baiden P, Asiedu AB (2011). Housing and health in three contrasting neighbourhoods in Accra, Ghana. Soc Sci Med.

[20] Boadi KO, Kuitunen M (2006). Factors affecting the choice of cooking fuel, cooking place and respiratory health in the Accra metropolitan area, Ghana. J Biosoc Sci.

[21] Fink G, Arku R, Montana L (2012). The health of the poor: women living in informal settlements. Ghana Med J.

[22] Jankowska MM, Weeks JR, Engstrom R (2012). Do the most vulnerable people live in the worst slums? A spatial analysis of Accra, Ghana. Ann GIS.

[23] Jankowska MM, Benza M, Weeks JR (2013). Estimating spatial inequalities of urban child mortality. Demogr Res.

[24] Stoler J, Weeks JR, Getis A, Hill AG (2009). Distance threshold for the effect of urban agriculture on elevated self-reported malaria prevalence in Accra, Ghana. Am J Trop Med Hyg.

[25] Weeks JR, Getis A, Stow DA, Hill AG, Rain D, Engstrom R (2012). Connecting the dots between health, poverty and place in Accra, Ghana. Ann Assoc Am Geogr.

[26] Weeks JR, Hill AG, Stoler J (2014). Spatial Inequalities: health, poverty, and place in Accra, Ghana.

[27] Eyles J, Wilson K, Mu L, Keller-Olaman S, Elliott S (2009). What people think about the environment and its relationship to their health: perceptions of health at different scales of environment in Hamilton, Ontario. Local Environ.

[28] Goldman N, Pebley AR, Beckett M (2001). Diffusion of ideas about personal hygiene and contamination in poor countries: evidence from Guatemala. Soc Sci Med.

[29] Andrzejewski CS, Reed HE, White MJ (2009). Does where you live influence what you know? Community effects on health knowledge in Ghana. Health Place.

[30] Scammell MK (2010). Qualitative environmental health research: an analysis of the literature, 1991–2008. Environ Health Perspect.

[31] Weber JM, Hair JF, Fowler CR (2000). Developing a measure of perceived environmental risk. J Environ Educ.

[32] Cargo M, Mercer SL (2008). The value and challenges of participatory research: strengthening its practice. Annu Rev Public Health.

[33] O'Fallon LR, Dearry A (2002). Community-based participatory research as a tool to advance environmental health sciences. Environ Health Perspect.

[34] Engstrom R, Rain D, Jewell H, Ofiesh C, Weeks JR (2013). Defining neighborhood boundaries for urban health research in developing countries: a case study of Accra, Ghana. J Maps.

[35] Hill AG, Darko R, Seffah J, Adanu RMK, Anarfi JK, Duda RB (2007). Health of urban Ghanaian women as identified by the Women's Health Study of Accra. Int J Gynaecol Obstet.

[36] The WHSA-II Writing Team (2011). Final report on the Women's Health Study of Accra Wave II.

[37] Darko R, Adanu RM, Duda RB, Douptcheva N, Hill AG (2012). The health of adult women in Accra, Ghana: self-reporting and objective assessments 2008–2009. Ghana Med J.

[38] Thomas DR (2006). A general inductive approach for analyzing qualitative evaluation data. Am J Eval.

[39] Songsore J, McGranahan G (1996). Women and household environmental care in the Greater Accra Metropolitan Area (GAMA), Ghana.

[40] UN-Habitat, Ghana Statistical Service (2003). The Accra Slum Survey of 2003.

[41] Klinkenberg E, McCall PJ, Wilson MD, Amerasinghe FP, Donnelly MJ (2008). Impact of urban agriculture on malaria vectors in Accra, Ghana. Malar J.

[42] Opoku AA, Ansa-Asare OD, Amoako J (2007). The occurrences and habitat characteristics of mosquitoes in Accra, Ghana. West African J Appl Ecol.

[43] Chinery WA (1984). Effects of ecological changes on the malaria vectors *Anopheles funestus* and the *Anopheles gambiae* complex of mosquitoes in Accra, Ghana. J Trop Med Hyg.

[44] Awolola TS, Oduola AO, Obansa JB, Chukwurar NJ, Unyimadu JP (2007). *Anopheles gambiae* s.s. breeding in polluted water bodies in urban Lagos, southwestern Nigeria. J Vector Borne Dis.

[45] Castro MC, Kanamori S, Kannady K, Mkude S, Killeen GF, Fillinger U (2010). The importance of drains for the larval development of lymphatic filariasis and malaria vectors in Dar es Salaam, United Republic of Tanzania. PLoS Negl Trop Dis.

[46] Sattler MA, Mtasiwa D, Kiama M, Premji Z, Tanner M, Killeen GF (2005). Habitat characterization and spatial distribution of *Anopheles* sp. mosquito larvae in Dar es Salaam (Tanzania) during an extended dry period. Malar J.

[47] Klinkenberg E, McCall PJ, Hastings IM, Wilson MD, Amerasinghe FP, Donnelly MJ (2005). Malaria and irrigated crops, Accra, Ghana. Emerg Infect Dis.

[48] Amexo M, Tolhurst R, Barnish G, Bates I (2004). Malaria misdiagnosis: effects on the poor and vulnerable. Lancet.

[49] 
Baba MM, Saron MF, Vorndam AV, Adeniji JA, Diop O, Olaleye D (2009). Dengue virus infections in patients suspected of malaria/typhoid in Nigeria. J Am Sci.

[50] O'Dempsey TJD, McArdle TF, Laurence BE, Lamont AC, Todd JE, Greenwood BM (1993). Overlap in the clinical features of pneumonia and malaria in African children. Trans R Soc Trop Med Hyg.

[51] Ye Y, Madise N, Ndugwa R, Ochola S, Snow RW (2009). Fever treatment in the absence of malaria transmission in an urban informal settlement in Nairobi, Kenya. Malar J.

[52] Stoler J, Al Dashti R, Anto F, Fobil J, Awandare GA (2014). Deconstructing ‘malaria’: West Africa as the next front for dengue fever surveillance and control. Acta Trop.

[53] Appawu M, Dadzie S, Abdul H, Asmah H, Boakye D, Wilson M (2006). Surveillance of viral haemorrhagic fevers in Ghana: entomological assessment of the risk of transmission in the northern regions. Ghana Med J.

[54] Chinery WA (1970). A survey of mosquito breeding in Accra, Ghana during a 2 year period of larval mosquito control. Part 3: The breeding of *Aedes* (Stegomyia) *aegypti*, Linnaeus, in Accra. Ghana Med J.

[55] Adongo PB, Kirkwood B, Kendall C (2005). How local community knowledge about malaria affects insecticide-treated net use in northern Ghana. Trop Med Int Health.

[56] Ayi I, Nonaka D, Adjovu JK, Hanafusa S, Jimba M, Bosompem KM (2010). School-based participatory health education for malaria control in Ghana: engaging children as health messengers. Malar J.

[57] Gazzinelli MFC, Kloos H, de Cássia Marques R, Dos Reis DC, Gazzinelli A (2008). Popular beliefs about the infectivity of water among school children in two hyperendemic schistosomiasis areas of Brazil. Acta Trop.

[58] Donkor ES, Tetteh-Quarcoo PB, Nartey P, Agyeman IO (2012). Self-medication practices with antibiotics among tertiary level students in Accra, Ghana: a cross-sectional study. Int J Environ Res Public Health.

[59] Smith F (2009). The quality of private pharmacy services in low and middle-income countries: a systematic review. Pharm World Sci.

[60] Smith F (2004). Community pharmacy in Ghana: enhancing the contribution to primary health care. Health Policy Plan.

[61] Scott B, Curtis V, Rabie T, Garbrah-Aidoo N (2007). Health in our hands, but not in our heads: understanding hygiene motivation in Ghana. Health Policy Plan.

[62] Arku RE, Vallarino J, Dionisio KL, Willis R, Choi H, Wilson JG (2008). Characterizing air pollution in two low-income neighborhoods in Accra, Ghana. Sci Total Environ.

[63] Songsore J, McGranahan G, Marcotullio P, McGranahan G (2007). Poverty and the environmental health agenda in a low-income city: the case of the Greater Accra Metropolitan Area (GAMA), Ghana. Scaling urban environmental challenges: from local to global and back.

[64] Zhou Z, Dionisio KL, Arku RE, Quaye A, Hughes AF, Vallarino J (2011). Household and community poverty, biomass use, and air pollution in Accra, Ghana. Proc Natl Acad Sci USA.

[65] Stoler J, Delimini RK, Bonney JHK, Oduro AR, Owusu-Agyei S, Fobil JN (2015). Evidence of recent dengue exposure among malaria parasite-positive children in three urban centers in Ghana. Am J Trop Med Hyg.

